# Supplementary material for: In situ photodeposition of platinum clusters on a covalent organic framework for photocatalytic hydrogen production
Source: Nat Commun. 2022 Mar 15;13:1355. doi: 10.1038/s41467-022-29076-z (PMC8924255; doi:10.1038/s41467-022-29076-z)
Supplement: Supplementary file 1 — Supplementary Information [file 41467_2022_29076_MOESM1_ESM.pdf]

Supplementary Information

In situ photodeposition of platinum clusters on a covalent organic framework for photocatalytic hydrogen production

Yimeng Li[1#], Li Yang[1#], Huijie He[1], Lei Sun[1], Honglei Wang[1], Xu Fang[1], Yanliang Zhao[1], Daoyuan Zheng[1], Yu Qi[2], Zhen Li[1]* and Weiqiao Deng[1,2]*

1、Institute of Molecular Sciences and Engineering, Institute of Frontier and Interdisciplinary Science, Shandong University, Qingdao, Shandong, 266237, China.

2、Dalian National Laboratory for Clean Energy, Dalian Institute of Chemical Physics, Chinese Academy of Sciences, Dalian 116023, China

* Corresponding author, E-mail: zhen_li@sdu.edu.cn; dengwq@sdu.edu.cn

[#] These authors contributed equally to this work.

Section I: supplementary Figures and Tables.

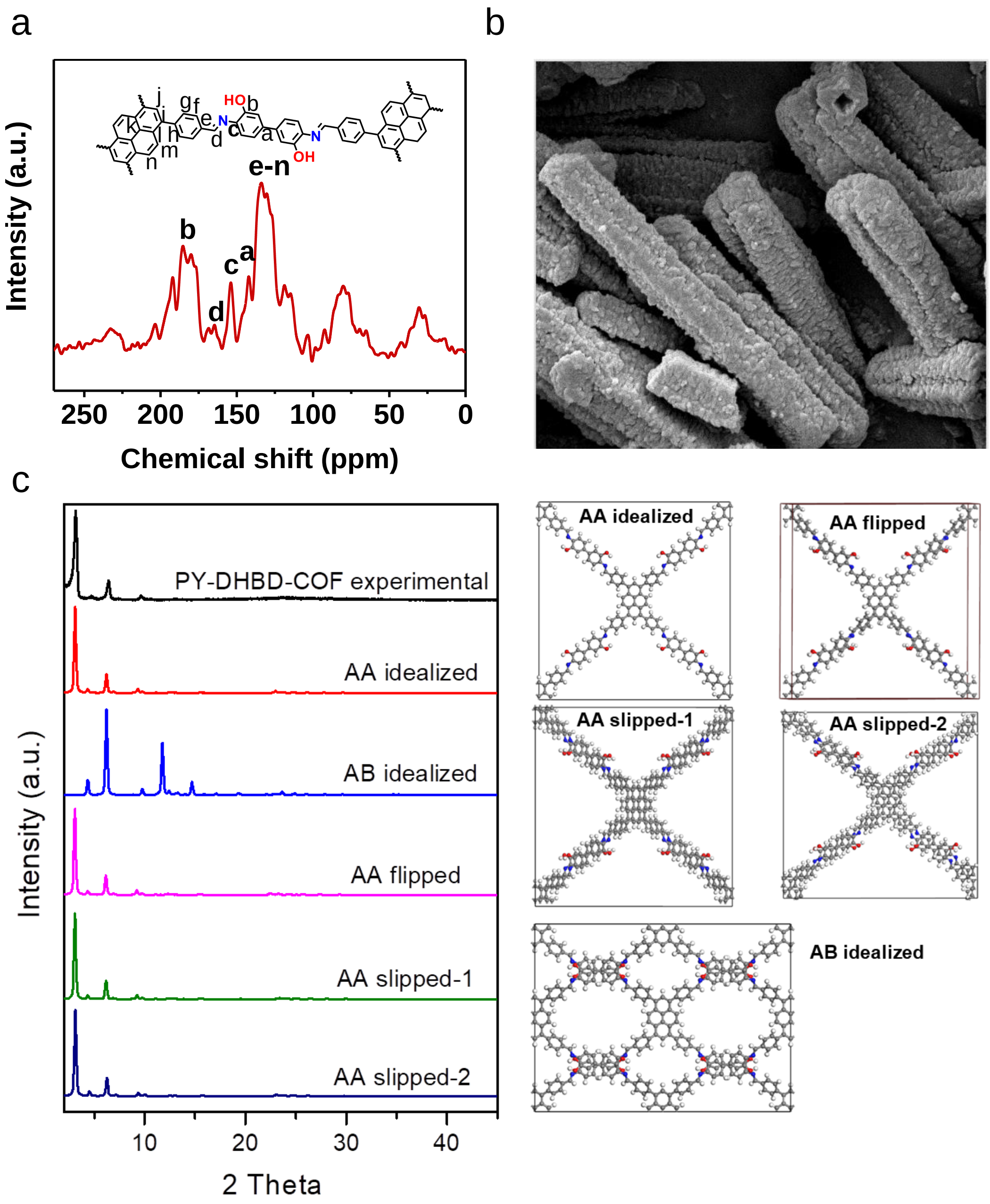

Supplementary Figure 1. Structural characterizations and morphology of PY-DHBD-COF. **(a)** Solid state ^{13}C-NMR spectrum of PY-DHBD-COF. **(b)** SEM image of PY-DHBD-COF. **(c)** Comparison of experimental and simulated powder X-ray diffraction patterns of several possible stacking models of PY-DHBD-COF including AA idealized, AB idealized, AA flipped, AA slipped-1 (with offset of 1.44 Å in the (110) direction) and AA slipped-2 (with offset of 2.88 Å in the (110) direction).

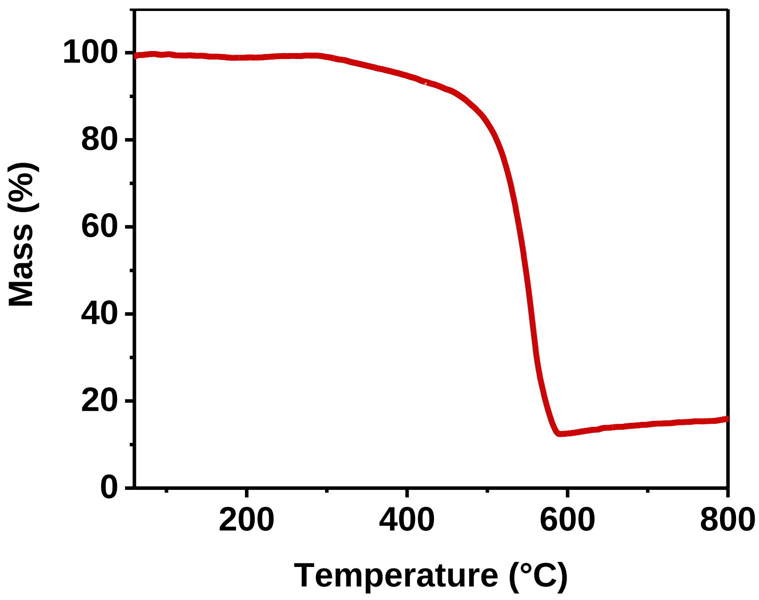

Supplementary Figure 2. TGA profile for PY-DHBD-COF.

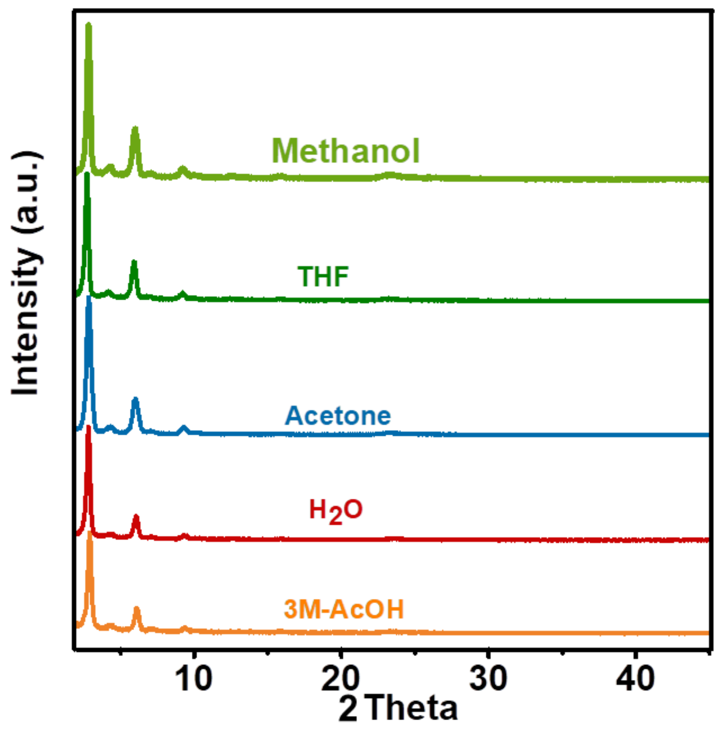

Supplementary Figure 3. PXRD patterns of the PY-DHBD-COF samples after soaking in difference solvents

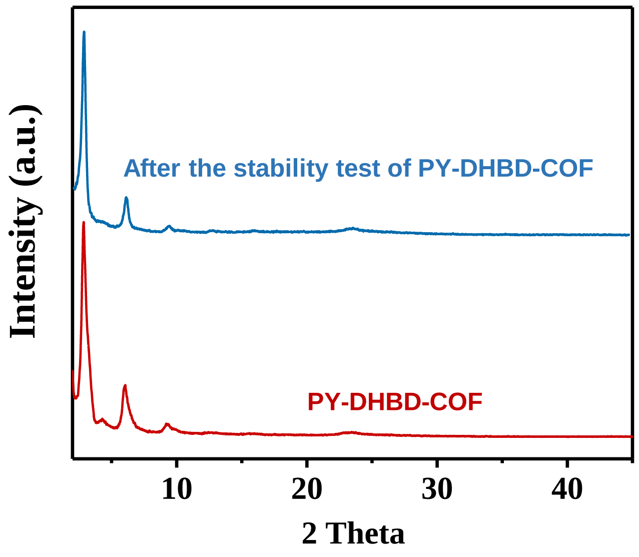

Supplementary Figure 4. PXRD patterns of PY-DHBD-COF before and after the long-term stability test.

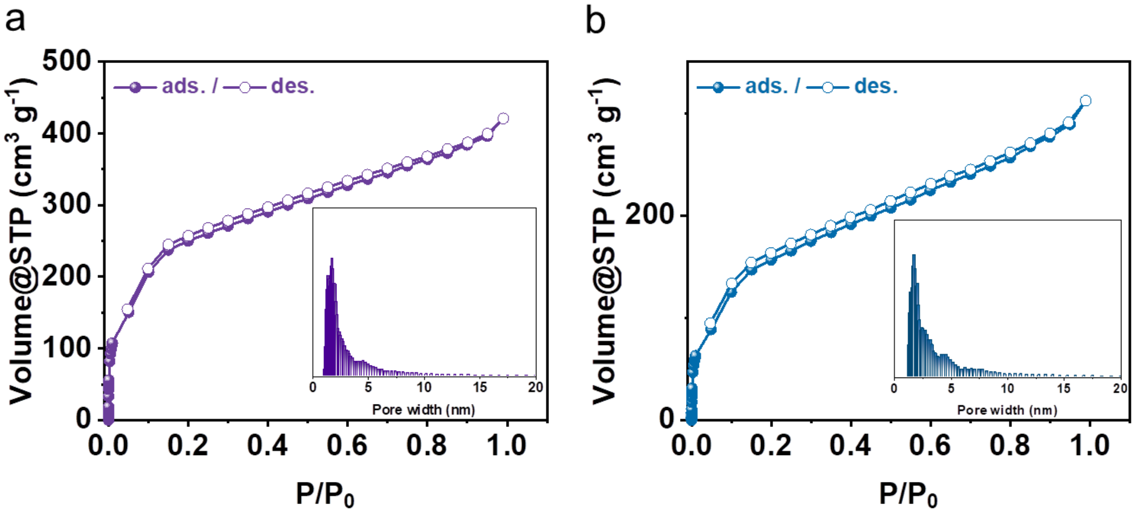

Supplementary Figure 5. N_2 adsorption isotherms of 3wt%-Pt-PY-DHBD-COF. (a) N_2 adsorption isotherms of 3wt%-Pt-PY-DHBD-COF before the long-term stability test. (b) N_2 adsorption isotherms of 3wt%-Pt-PY-DHBD-COF after the long-term stability test. Insert: pore size distributions.

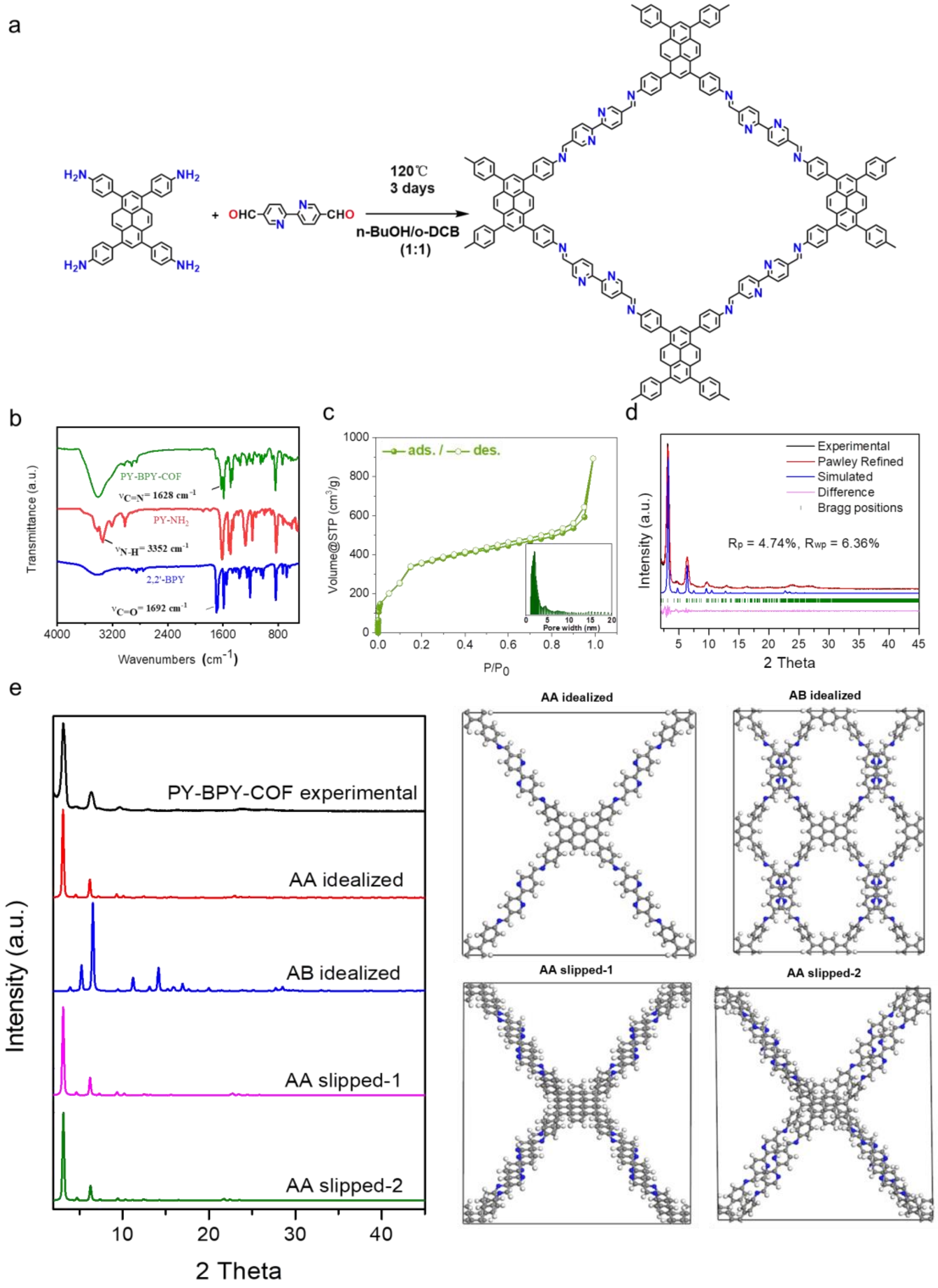

Supplementary Figure 6. Synthesis and characterizations of PY-BPY-COF. (a) Synthesis process and structure of PY-BPY-COF. (b) FT-IR spectra of PY-BPY-COF and the building monomers. (c) N_2 adsorption isotherms of PY-BPY-COF. (d) Experimental and simulated PXRD patterns of PY-BPY-COF. (e) Comparison of experimental and simulated powder X-ray

diffraction patterns of several possible stacking models of PY-BPY-COF including AA idealized, AB idealized, AA slipped-1 (with offset of 1.44 Å in the (110) direction) and AA slipped-2 (with offset of 2.88 Å in the (110) direction).

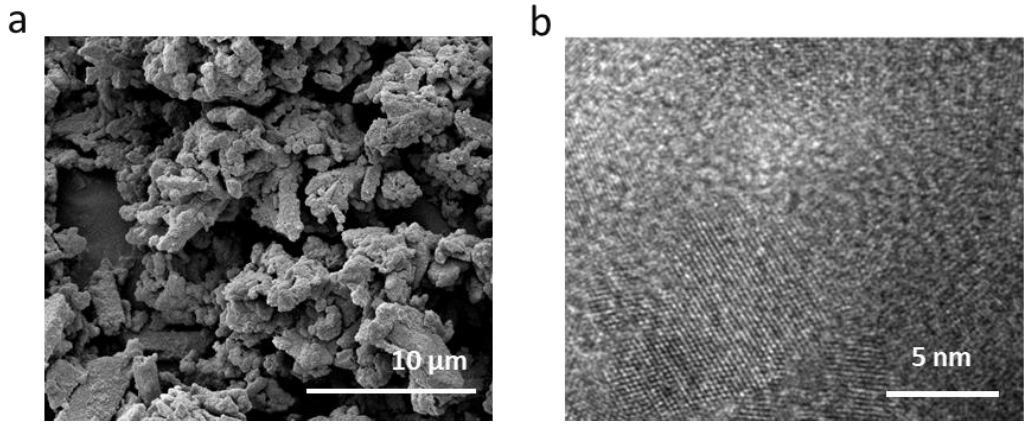

Supplementary Figure 7. Morphologies of PY-BPY-COF. (a) SEM images of PY-BPY-COF. (b) HR-TEM images of PY-BPY-COF.

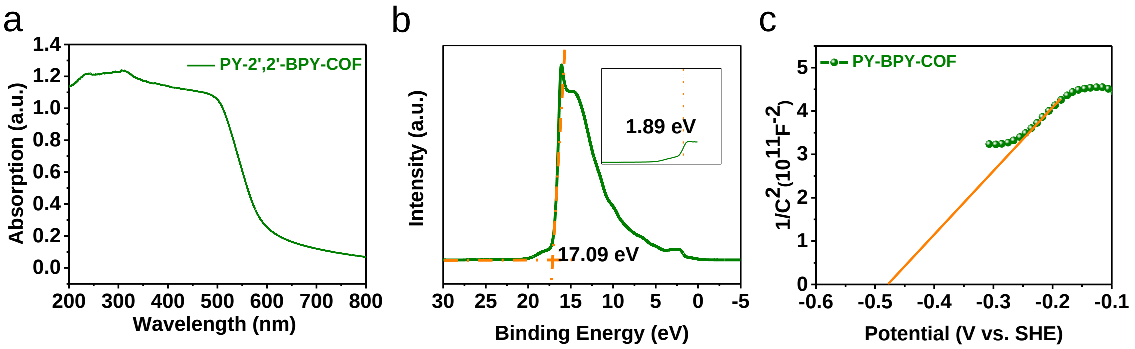

Supplementary Figure 8. Optical and electronic properties of PY-BPY-COF. (a) UV-Vis diffuse reflectance spectrum of PY-BPY-COF. (b) UPS spectrum of PY-BPY-COF. (c) Mott-Schottky plot of PY-BPY-COF.

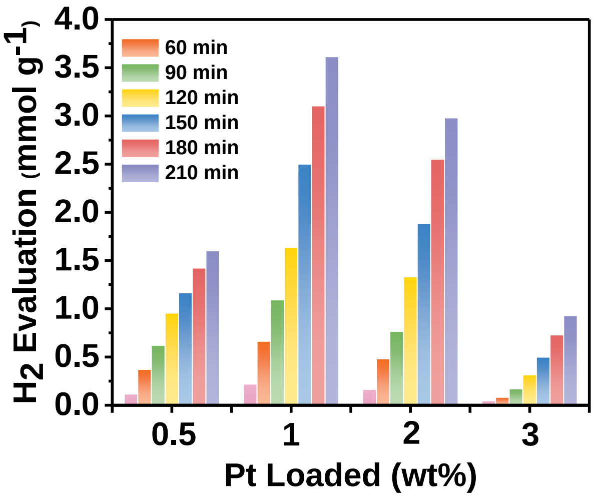

Supplementary Figure 9. Time dependent hydrogen evolution for PY-BPY-COF with different Pt loading amount. (10 mg catalyst was dispersed in 100 mL water, 10 mM ascorbic acid as electron donor, H_2PtCl_6 (0.376 g Pt L^{-1}) as Pt precursor, 300 W Xe lamp, λ>420 nm).

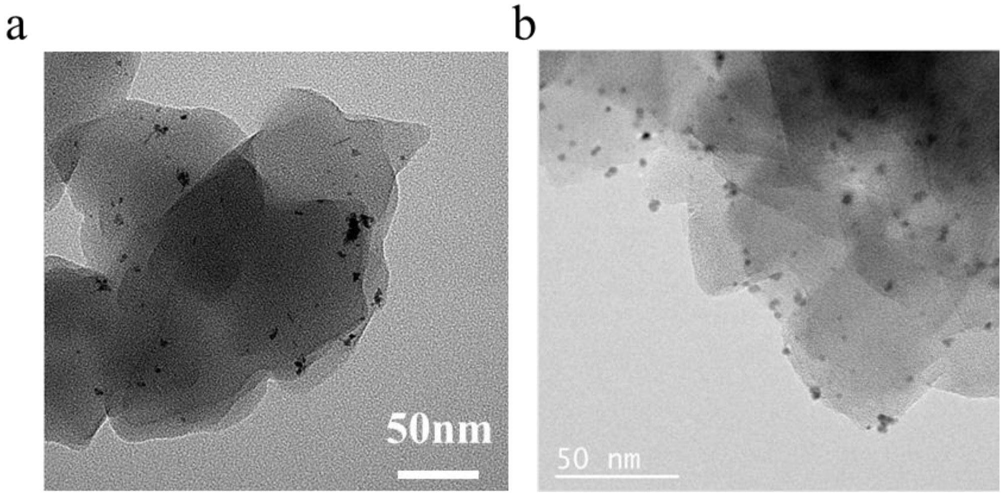

Supplementary Figure 10. TEM images of Pt-PY-BPY-COF. (a) 0.5 wt% Pt loading. (b) 1 wt% Pt loading.

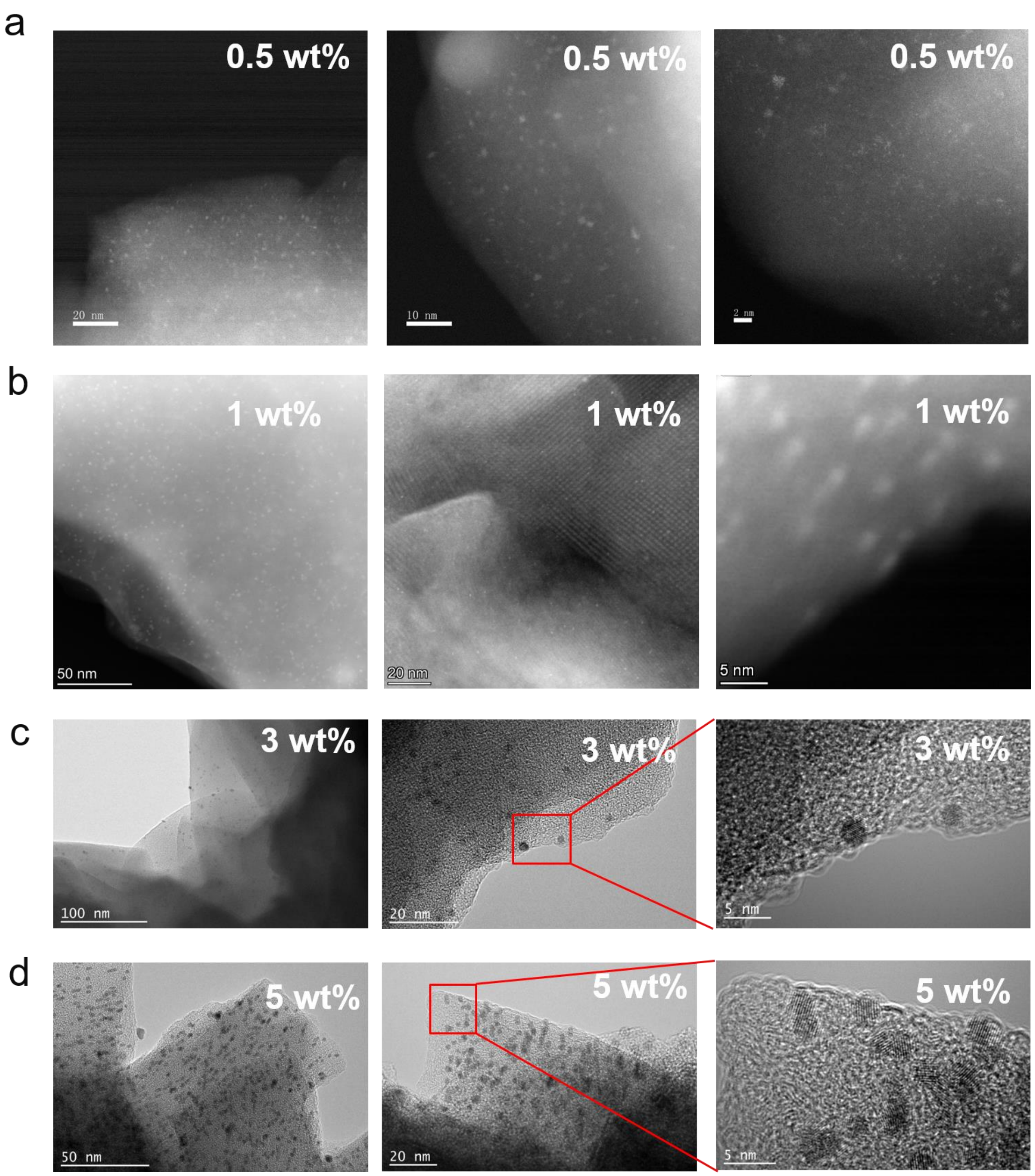

Supplementary Figure 11. HAADF-STEM and HR-TEM images of Pt-PY-DHBD-COF. (a) 0.5 wt% Pt loading. (b) 1 wt% Pt loading. (c) 3 wt% Pt loading. (d) 5 wt% Pt loading.

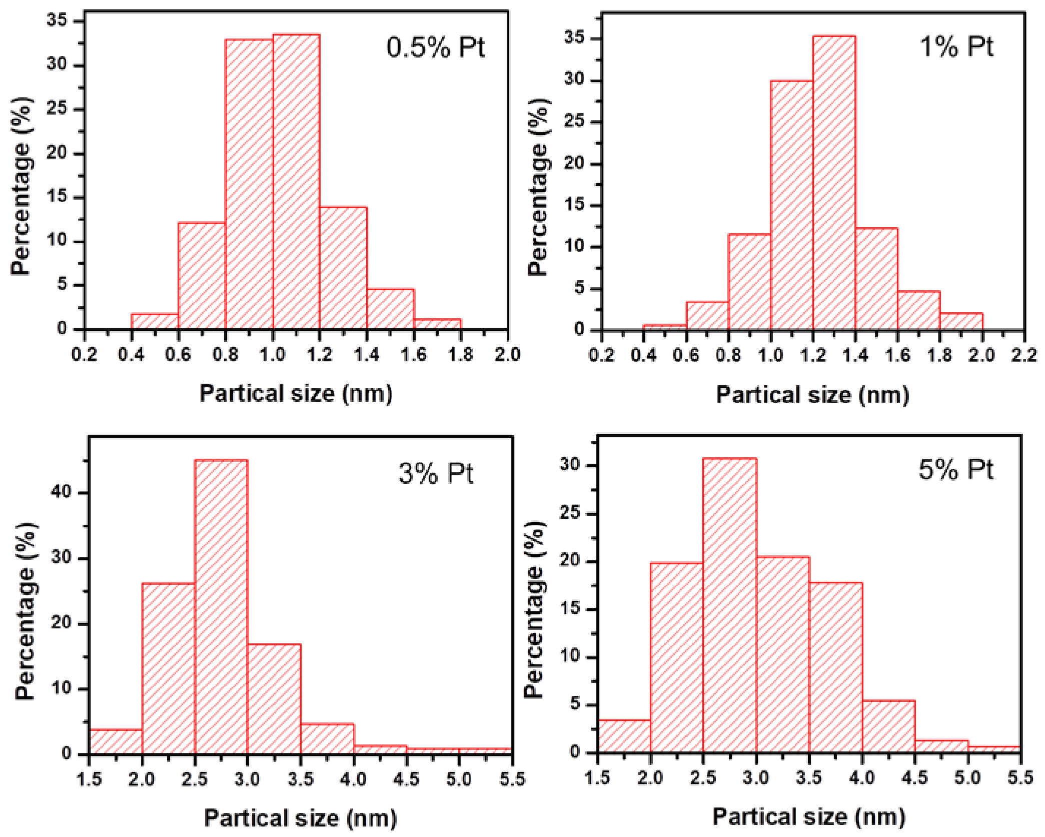

Supplementary Figure 12. The diameter distributions of Pt on PY-DHBD-COF with different Pt loading.

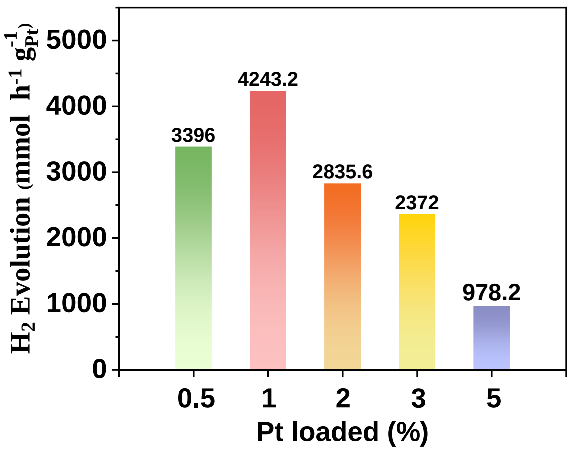

Supplementary Figure 13. HER activity normalized according to the loading amount of Pt.

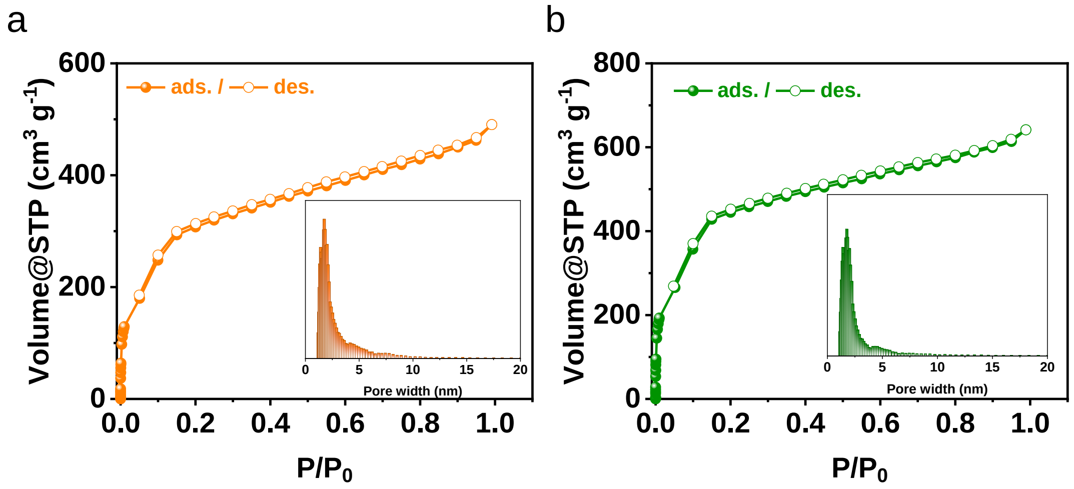

Supplementary Figure 14. N_2 adsorption isotherms of typical samples. (a) 1%wt Pt loaded PY-DHBD-COF. (b) Light-treated PY-DHBD-COF. PY-DHBD-COF was irradiated under the same photodepostion condition except for the existence of Pt precursor H_2PtCl_6 in the solution.

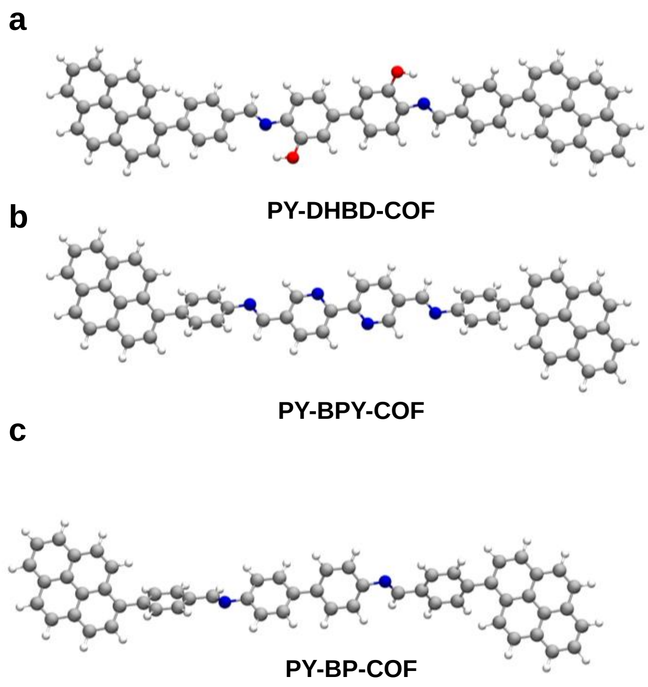

Supplementary Figure 15. The simplified COFs fragment model. (a) PY-DHBD-COF. (b) PY-BPY-COF. (c) PY-BP-COF.

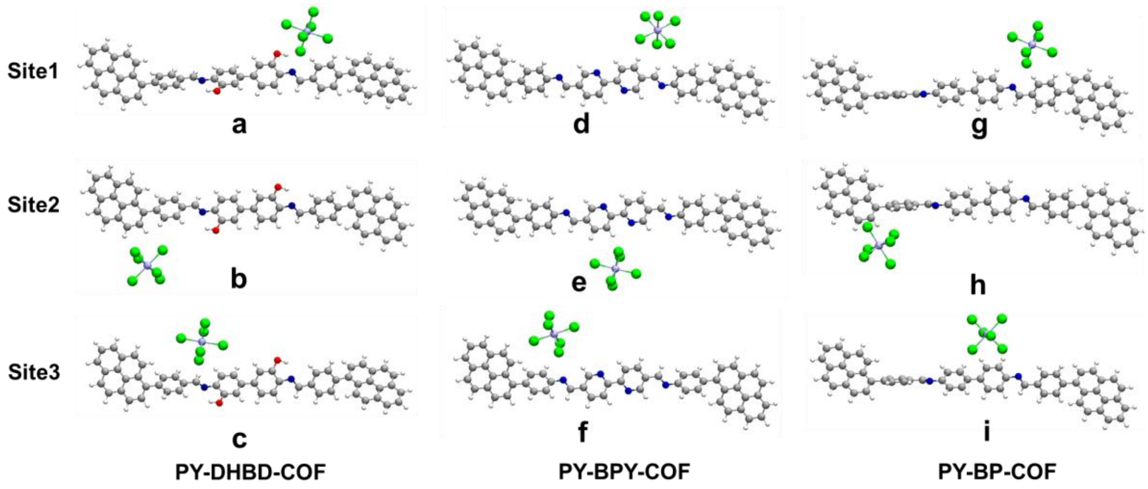

Supplementary Figure 16. The optimized configurations of $PtCl_6^{2-}$ on COFs. (a-c) PY-DHBD-COF, (d-f) PY-BPY-COF and (g-i) PY-BP-COF.

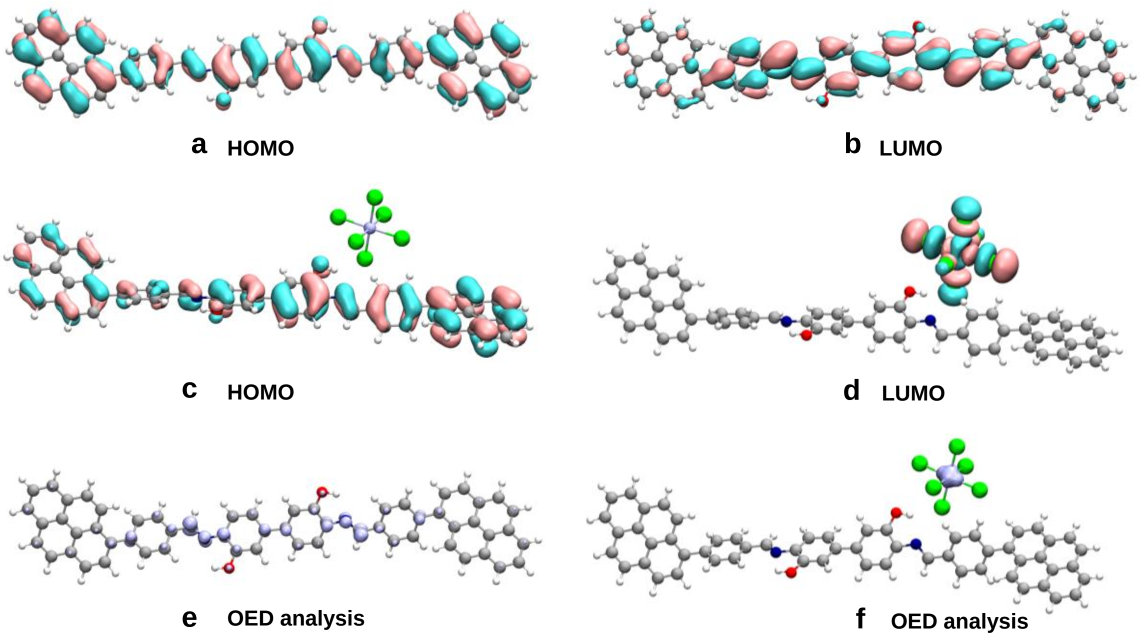

Supplementary Figure 17. The orbital and odd electron density analysis. The HOMO and LUMO orbital distribution on the fragment of PY-DHBD-COF (a and b) and the complex PY-DHBD-COF-$PtCl_6^{2-}$ (c and d). The odd electron density analysis based on the TD-DFT calculation of PY-DHBD-COF (e) and the complex PY-DHBD-COF-$PtCl_6^{2-}$ (f).

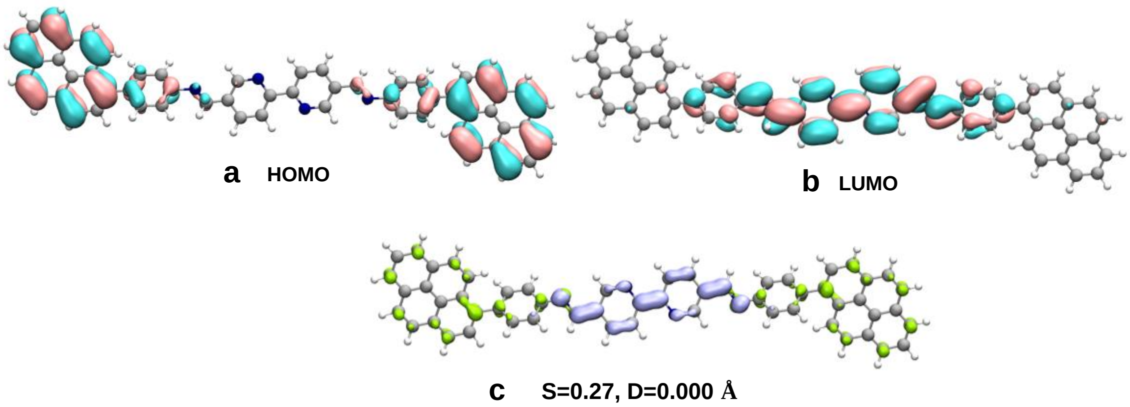

Supplementary Figure 18. The orbital and hole-electron analysis of PY-BPY-COF. The HOMO (a) and LUMO (b) orbital distribution on the construction fragment of PY-BPY-COF. (c)The hole (lime) and electron (violet) distribution of S1 excited state on PY-BPY-COF. S is the overlap integral of hole-electron distribution and D means the distance between centroid of hole and electron.

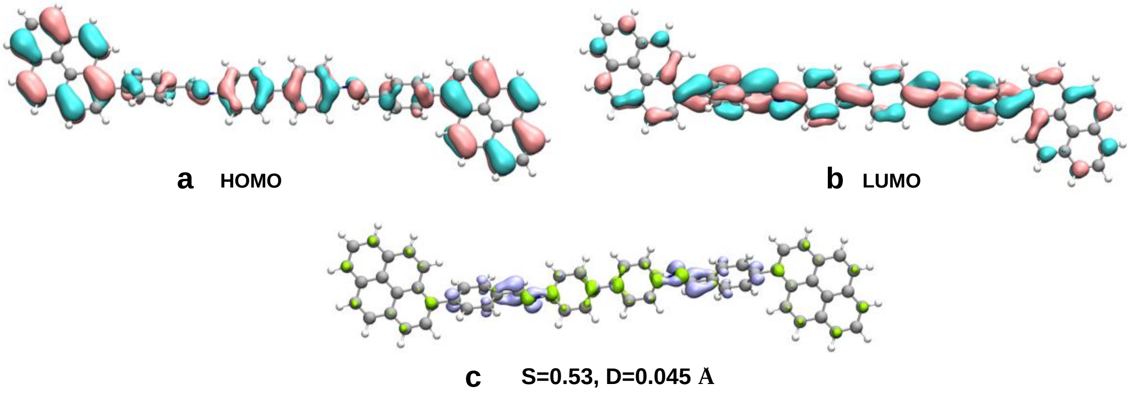

Supplementary Figure 19. The orbital and hole-electron analysis of PY-BP-COF. The HOMO (a) and LUMO (b) orbital distribution on the construction fragment of PY-BP-COF. (c) The hole (lime) and electron (violet) distribution of S1 excited state on PY-BP-COF. S is the overlap integral of hole-electron distribution and D means the distance between centroid of hole and electron.

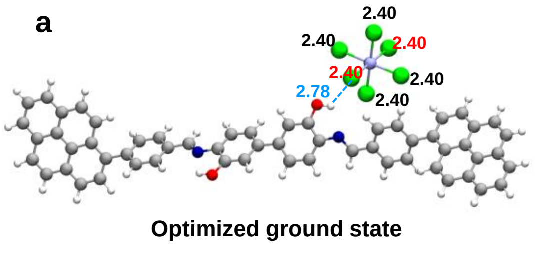

Supplementary Figure 20. The full optimized configurations of PY-DHBD-COF-$PtCl_6^{2-}$ under different states. (a) Ground state. (b) S1 excited state.

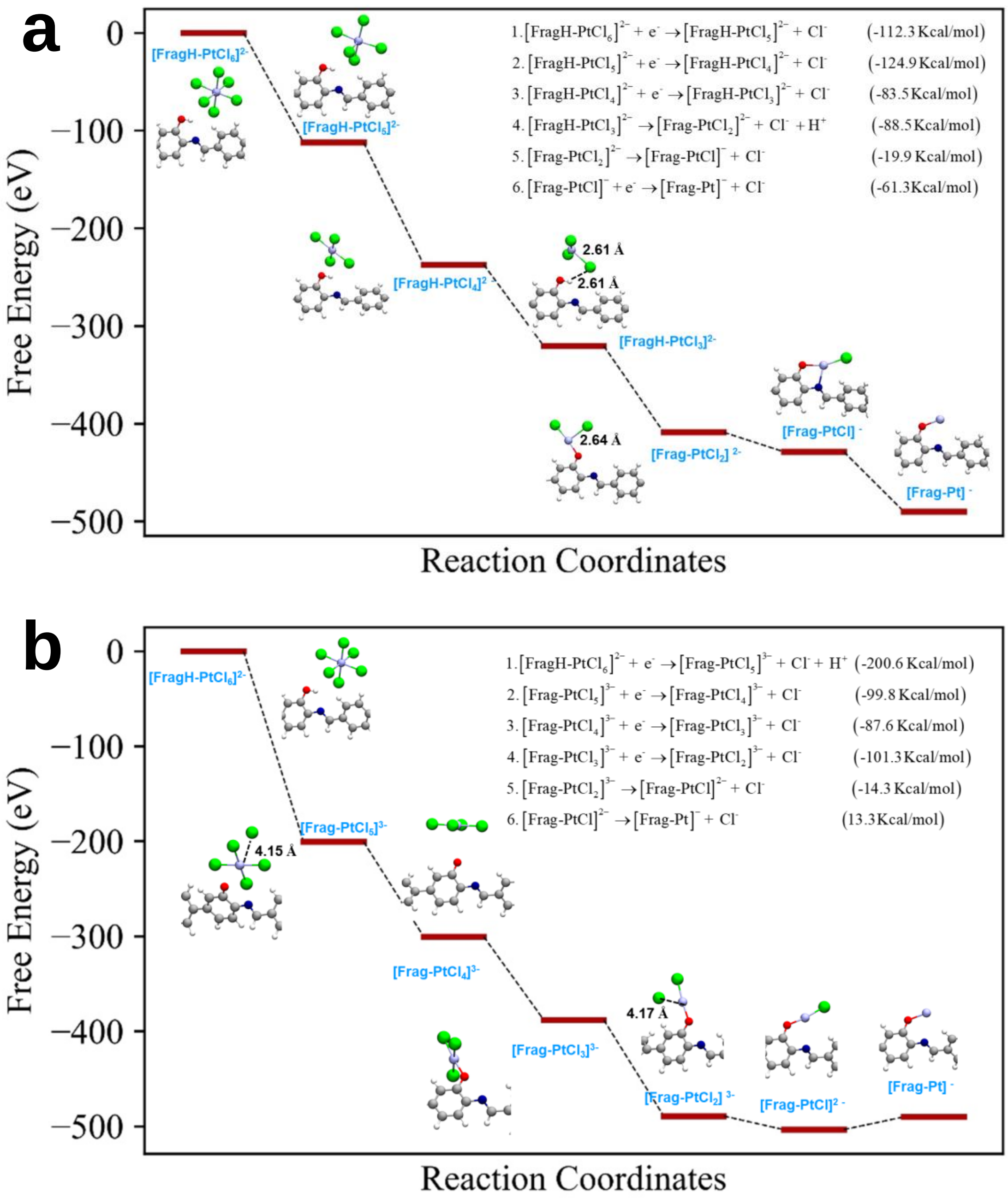

Supplementary Figure 21 Free energy diagrams of two possible photodeposition reaction paths. (a) The hydroxyls are deprotonated at the forth step, (b) the hydroxyls are deprotonated at the first step.

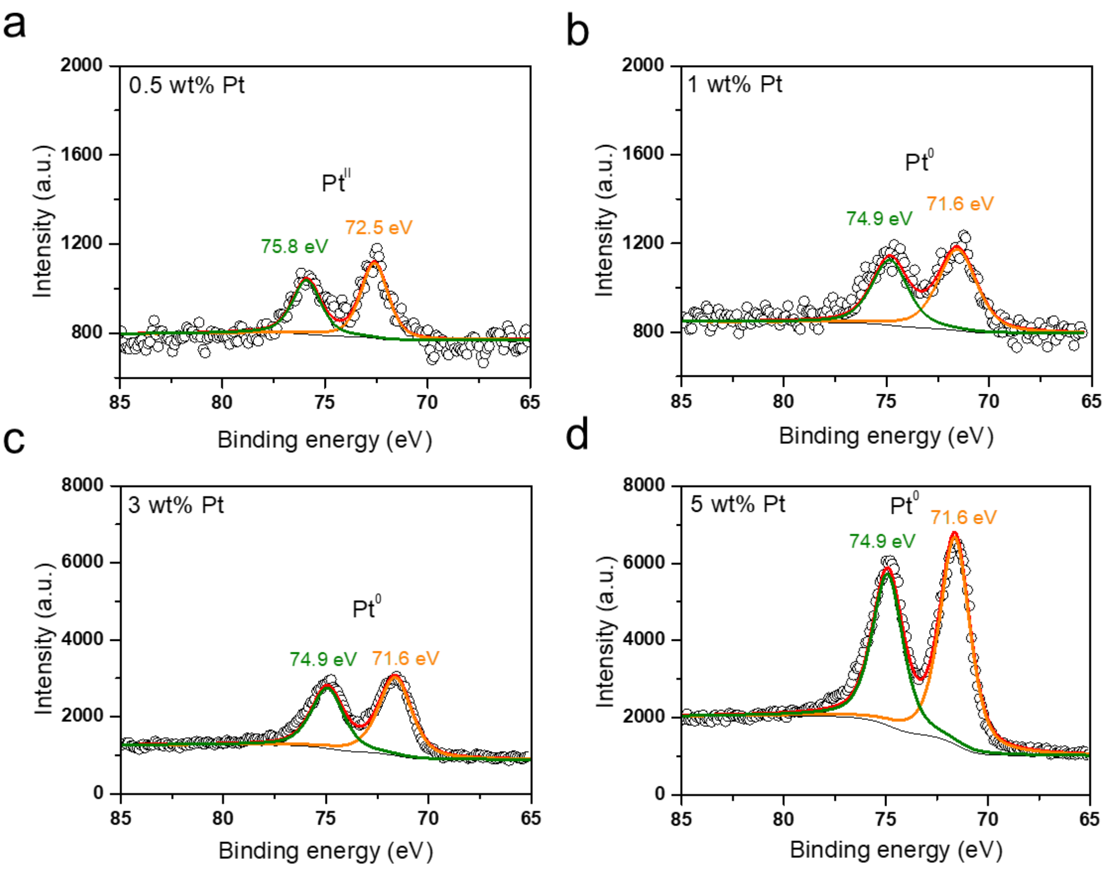

Supplementary Figure 22. XPS spectra of Pt 4*f* in PY-DHBD-COF with different Pt loading amounts. (a) 0.5 wt% Pt loading. (b) 1 wt% Pt loading. (c) 3 wt% Pt loading. (d) 5 wt% Pt loading.

Supplementary Table 1. Stacking distance and stacking energy for different periodic PY-DHBD-COF stacking models.

Stacking models	Stacking distance (Å)	Total staking Energy (Kcal/mol)	vdW stacking Energy (Kcal/mol)
Idealized	3.82	-309.6	-306.3
Flipped	3.97	-287.8	-284.6
Slipped-1	3.83	-304.9	-301.6
Slipped-2	3.89	-300.0	-296.6

Supplementary Table 2. Long-term hydrogen production for 3 wt% Pt loaded PY-DHBD-COF.

Term 1		Term 2		Term 3	
Time （h）	H_2 Envoluation (mmol g^{-1})	Time （h）	H_2 Envoluation (mmol g^{-1})	Time （h）	H_2 Envoluation (mmol g^{-1})
0.0	0.0	7.5	0.0	12.5	0.0
0.5	52.1	8.0	51.5	13.0	57.9
1.0	76.0	8.5	78.4	13.5	90.9
1.5	87.5	9.0	104.3	14.0	120.2
2.0	106.3	9.5	128.6	14.5	147.6
2.5	120.8	10.0	151.7	15.0	172.1
3.0	134.3	10.5	172.3	15.5	194.8
3.5	146.9	11.0	191.3	16.0	210.2
4.0	158.6	11.5	206.3	16.5	218.9
4.5	169.4	12.0	211.6	17.0	219.7
5.0	178.6	12.5	211.7	17.5	216.2
5.5	187.8				
6.0	195.6				
6.5	203.0				
7.0	210.2				
7.5	215.7				

Term 4		Term 5		Term 6	

Time （h）	H_2 Envoluation (mmol g^{-1})	Time （h）	H_2 Envoluation (mmol g^{-1})	Time （h）	H_2 Envoluation (mmol g^{-1})
17.5	0.0	22.5	0.0	27.5	0.0
18.0	60.3	23.0	57.0	28.0	54.4
18.5	90.3	23.5	92.4	28.5	84.7
19.0	120.1	24.0	121.8	29.0	111.3
19.5	146.8	24.5	148.4	29.5	135.1
20.0	172.0	25.0	171.1	30.0	153.0
20.5	193.0	25.5	191.1	30.5	175.8
21.0	212.8	26.0	209.1	31.0	188.5
21.5	225.5	26.5	222.6	31.5	212.2
22.0	227.0	27.0	221.6	32.0	212.3
22.5	226.2	27.5	221.2	32.5	212.4

Term 7		Term 8		Term 9	
Time （h）	H_2 Envoluation (mmol g^{-1})	Time （h）	H_2 Envoluation (mmol g^{-1})	Time （h）	H_2 Envoluation (mmol g^{-1})
32.5	0.0	37.5	0.0	42.5	0.0
33.0	55.9	38.0	52.5	43.0	48.3
33.5	90.6	38.5	81.4	43.5	74.7
34.0	122.5	39.0	117.4	44.0	100.2
34.5	148.3	39.5	138.6	44.5	124.0
35.0	171.3	40.0	157.1	45.0	146.4
35.5	190.5	40.5	174.9	45.5	166.4
36.0	202.3	41.0	188.2	46.0	186.2
36.5	207.0	41.5	210.0	46.5	203.3
37.0	207.7	42.0	216.0	47.0	206.5
37.5	207.9	42.5	216.1	47.5	207.3

Term 10		Term 11		Term 12	
Time	H_2 Envoluation	Time	H_2 Envoluation	Time	H_2 Envoluation

（h）	(mmol g^{-1})	（h）	(mmol g^{-1})	（h）	(mmol g^{-1})
47.5	0.0	52.5	0.0	57.5	0.0
48.0	50.9	53.0	59.2	58.0	50.9
48.5	80.2	53.5	81.8	58.5	80.2
49.0	105.7	54.0	103.8	59.0	106.7
49.5	130.4	54.5	127.0	59.5	130.4
50.0	147.9	55.0	144.2	60.0	147.9
50.5	166.0	55.5	161.2	60.5	166.1
51.0	188.1	56.0	176.7	61.0	188.1
51.5	197.3	56.5	191.8	61.5	197.4
52.0	199.0	57.0	213.7	62.0	199.0
52.5	206.5	57.5	214.6	62.5	206.5

Supplementary Table 3. Porosity parameters of PY-DHBD-COF and 1wt%-Pt-PY-DHBD-COF.

Samples	Surface area (m^2 g^{-1})	Pore volume (cm^3 g^{-1})
[a]PY-DHBD-COF$_{(original)}$	1893	0.918
[b]PY-DHBD-COF$_{(light\text{-}treated)}$	1603	0.874
[c]1wt%-Pt-PY-DHBD-COF	1106	0.663
[d]3wt%-Pt-PY-DHBD-COF$_{(before)}$	900	0.568
[e]3wt%-Pt-PY-DHBD-COF$_{(after)}$	571.4	0.416

[a] PY-DHBD-COF sample was the original material without any treatment.

[b] The PY-DHBD-COF$_{(light\text{-}treated)}$ sample was treated under a photodepostion condition (10 mg sample in 100 mL water, 10 mM ascorbic acid, 300 W Xe lamp, λ > 420 nm, light irradiation for 1.5 h) except for the existence of Pt precursor H_2PtCl_6 in the solution.

[c,d] The 1wt%-Pt-PY-DHBD-COF and 3wt%-Pt-PY-DHBD-COF$_{(before)}$ were obtained under the photodepostion condition (10 mg catalyst in 100 mL water, 10 mM ascorbic acid, H_2PtCl_6 (0.376 g Pt L^{-1}) as precursor, 300 W Xe lamp, λ > 420 nm, light irradiation for 1.5 h).

[e] The 3wt%-Pt-PY-DHBD-COF$_{(after)}$ was the sample recovered after the long-term hydrogen evolution of 60 h.

Supplementary Table 4. The photocatalytic hydrogen evolution performance comparison of PY-DHBD-COF with other representative COF and MOF based photocatalysts.

Entry	Co-catalyst	Sacrificial reagent	Light Source	HER (μmol g^{-1} h^{-1})	AQY (at 420 nm) (%)	Ref.
PY-DHBD-COF	Pt 0.5wt%	Ascorbic acid	> 420 nm	16980	--	This work
PY-DHBD-COF	Pt 1wt%	Ascorbic acid	> 420 nm	42432	--	This work
PY-DHBD-COF	Pt 2wt%	Ascorbic acid	> 420 nm	56712	--	This work
PY-DHBD-COF	Pt 3wt%	Ascorbic acid	> 420 nm	71160	8.4	This work
PY-DHBD-COF	Pt 5wt%	Ascorbic acid	> 420 nm	48912	--	This work
Tp-2C/BPy^{2+}-COF (19.10%)	Pt 3wt%	Ascorbic acid	> 420 nm	34600	6.93	3
Py-ClTP-BT-COF	Pt 5wt%	Ascorbic acid	> 420 nm	8875	8.45	4
FS-COF	Pt 8wt%	Ascorbic acid	> 420 nm	10100	3.2	5
g-$C_{18}N_3$-COF	Pt 3wt%	Ascorbic acid	> 420 nm	2920	4.84	6
MIL-125/Au	Pt 0.49wt%	TEOA	> 420 nm	1743	--	7
Uio-66-NH_2	Pt 0.65wt%	Ascorbic acid	> 420 nm	1528	2.3	8
MIL-125-NH_2	Pt 0.45wt%	TEOA	> 420 nm	707		9
NU-100	Pt 1wt%	TEOA	> 400 nm	610	--	10

Supplementary Table 5. Adsorption energy (ΔE_{ads}) of $PtCl_6^{2-}$ on COFs.

COFs	Configurations	ΔE_{ads}（Kcal/mol）
**PY-DHBD-COF**	Site1	-8.96
	Site2	-5.25
	Site3	-8.15
**PY-BPY-COF**	Site1	-6.72
	Site2	-5.41
	Site3	-6.17
**PY-BP-COF**	Site1	-5.83
	Site2	-5.40
	Site3	-5.27

Supplementary Table 6. Vertical excitation energies for the PY-DHBD-COF.

Excitation	Excitation Energy (nm)	Oscillator Strength (a.u.)	Occupied Orbital	Virtual Orbital	2*|coeff.|^2 *100 (%)
S0-S1	580.14	3.8353	HOMO	LUMO	91.68
S0-S2	498.70	0.0065	HOMO-1	LUMO	83.52
S0-S3	477.03	0.0024	HOMO	LUMO+1	83.62
S0-S4	438.07	0.1863	HOMO-1	LUMO+1	69.79
S0-S5	435.73	0.2564	HOMO-2	LUMO	67.13

Supplementary Table 7. Vertical excitation energies for the PY-BPY-COF.

Excitation	Excitation Energy (nm)	Oscillator Strength (a.u.)	Occupied Orbital	Virtual Orbital	2*|coeff.|^2 *100 (%)
S0-S1	454.46	1.3234	HOMO	LUMO	90.15
S0-S2	441.60	0.0000	HOMO-1	LUMO	96.02
S0-S3	380.06	1.7055	HOMO-2	LUMO	74.07
S0-S4	362.85	0.0000	HOMO	LUMO+1	52.83
S0-S5	353.69	0.4774	HOMO-1	LUMO+1	58.31

Supplementary Table 8. Vertical excitation energies for the PY-BP-COF.

Excitation	Excitation Energy (nm)	Oscillator Strength (a.u.)	Occupied Orbital	Virtual Orbital	2*|coeff.|^2 *100 (%)
S0-S1	411.58	2.8802	HOMO	LUMO	71.04
S0-S2	390.87	0.0064	HOMO-1	LUMO	64.01
S0-S3	367.29	0.1342	HOMO-2	LUMO	71.57
S0-S4	361.22	0.0038	HOMO	LUMO+1	48.22
S0-S5	349.53	0.2295	HOMO-1	LUMO+1	67.93

Supplementary Table 9. The electron energies[1], zero point energy, entropies[2] and free energies[3] of all species in this work.

COFs	Complex	E_{ele} (Hartree)	*ZPE* (Hartree)	*S* (Cal/mol·K)	*G* (Hartree)
**PY-DHBD-COF**	FragH	-2491.985653	0.772007	281.147	-2491.300448
	FragH-$PtCl_6$-Site1	-5373.069177	0.780492	347.342	-5372.393677
	FragH-$PtCl_6$-Site2	-5373.063261	0.780483	351.005	-5372.38941
	FragH-$PtCl_6$-Site3	-5373.06788	0.780807	348.983	-5372.39284
	FragH-$PtCl_6$-Site1 -TD-OPT[2]	-5373.045082	0.780499	346.313	-5372.362381
	$[FragH\text{-}PtCl_5]^{2-}$	-4912.851211	0.778526	341.242	-4912.17621
	$[FragH\text{-}PtCl_4]^{2-}$	-4452.652669	0.777221	337.164	-4451.978728
	$[FragH\text{-}PtCl_3]^{2-}$	-3992.388356	0.775336	331.538	-3991.71507
	$[Frag\text{-}PtCl_2]^{2-}$	-3531.536765	0.762394	314.66	-3530.87114
	$[Frag\text{-}PtCl]^{-}$	-3071.178655	0.762492	295.203	-3070.506399
	Frag-Pt	-2610.877517	0.760441	292.796	-2610.207632
	$[Frag\text{-}PtCl_5]^{3-}$	-4912.384668	0.764739	353.142	-4911.728436
	$[Frag\text{-}PtCl_4]^{3-}$	-4452.152872	0.763782	333.556	-4451.490956
	$[Frag\text{-}PtCl_3]^{3-}$	-3991.897801	0.761105	318.787	-3991.234046
	$[Frag\text{-}PtCl_2]^{3-}$	-3531.661523	0.761857	320.86	-3530.999065
	$[Frag\text{-}PtCl]^{2-}$	-3071.292944	0.761388	303.758	-3070.625344
**PY-BPY-COF**	FragH	-2373.610474	0.739592	268.176	-2372.953892
	FragH-$PtCl_6$-Site1	-5254.690428	0.74793	342.986	-5254.04757
	FragH-$PtCl_6$-Site2	-5254.688343	0.747928	338.781	-5254.043578
	FragH-$PtCl_6$-Site3	-5254.689549	0.747929	336.259	-5254.043667
**PY-BP-COF**	FragH	-2341.533935	0.763474	267.153	-2340.852832
	FragH-$PtCl_6$-Site1	-5222.612471	0.771582	339.928	-5221.944321
	FragH-$PtCl_6$-Site2	-5222.61179	0.771548	340.73	-5221.943832
	FragH-$PtCl_6$-Site3	-5222.611572	0.771864	340.471	-5221.943698

[1] The electron energy, thermal correction to Gibbs free energy is calculated at the B3LYP-D3/6-31G* (C, N, O, H, Cl) & SDD+ECP (Pt) level.

[2] The TD-DFT optimization is conducted at the TD-B3LYP-D3/6-31G* (C, N, O, H, Cl) & SDD+ECP (Pt) level, and TD-DFT single point analysis is conducted at the TD-PBE0-D3/6-31G* (C, N, O, H, Cl) & SDD+ECP (Pt) level

Supplementary Table 10. Fractional atomic coordinates for the unit cell of PY-DHBD-COF.

Space group: C222<br>A = 39.9494 Å, b = 40.8391 Å, c = 3.8138 Å<br>α = β = γ = 90°			
Atom	x	y	z
C1	0.53076	0.42807	-0.04262
C2	0.53067	0.46500	-0.01729
C3	0.06044	0.98309	0.00352
C4	0.56014	0.59113	0.11016
C5	0.56359	0.62334	-0.02418
C6	0.58656	0.57926	0.31972
C7	0.59231	0.64195	0.03934
C8	0.61597	0.59716	0.36850
C9	0.61919	0.62874	0.22955
N10	0.65174	0.67775	0.17170
C11	0.67929	0.69735	0.18291
C12	0.21120	0.18542	0.27537
C13	0.67598	0.73068	0.09068
C14	0.23901	0.20608	0.27493
C15	0.70385	0.75138	0.10097
C16	0.73588	0.73951	0.19002
H17	0.91539	0.97169	-0.02786
H18	0.54526	0.63392	-0.19910
H19	0.08436	0.05644	0.45837
H20	0.59388	0.66618	-0.07490
H21	0.13579	0.08663	0.52651
H22	0.26280	0.19586	0.35517
O23	0.64533	0.74422	-0.01280
H24	0.70038	0.77658	0.02062
C25	0.64887	0.64628	0.28199
H26	0.21535	0.16008	0.34267
H27	0.33089	0.63445	-0.42118
H28	0.37408	0.72831	0.02029
C29	0.50000	0.41187	0.00000
C30	0.50000	0.48240	-0.00000
H31	0.50000	0.38558	0.00000

Supplementary Table 11. Fractional atomic coordinates for the unit cell of PY-BPY-COF.

Space group: P222<br>A = 36.9164 Å, b = 41.8713 Å, c = 3.9555 Å<br>α = β = γ = 90°			
Atom	x	y	z
C1	0.92294	0.97077	-0.03352
C2	0.96165	0.97097	-0.01253
C3	0.98154	0.94227	0.00083
H4	0.96805	0.08105	-0.01012
C5	0.09841	0.94272	0.07938
C6	0.13288	0.93925	-0.08575
C7	0.08783	0.91684	0.29622
C8	0.15390	0.91249	-0.04448
H9	0.14247	0.95802	-0.25873
C10	0.10856	0.88977	0.32667
H11	0.06267	0.91863	0.44388
C12	0.14133	0.88797	0.15825
H13	0.17972	0.91059	-0.18053
H14	0.10002	0.86987	0.48976
N15	0.16094	0.86075	0.20165
C16	0.19668	0.85479	0.08489
H17	0.21311	0.87313	-0.04195
C18	0.21095	0.82302	0.14195
C19	0.19092	0.79877	0.29843
C20	0.24653	0.81521	0.03883
C21	0.20600	0.76913	0.34174
H22	0.16316	0.80293	0.38809
N23	0.26223	0.78654	0.08286
H24	0.26433	0.83246	-0.08743
C25	0.24191	0.76357	0.23078
H26	0.19089	0.74974	0.46501
C27	0.42310	0.47120	-0.06195
C28	0.46167	0.47111	-0.02842
C29	0.48157	0.44238	-0.01084
H30	0.46806	0.58096	0.01063
C31	0.59774	0.44363	0.15048

C32	0.63370	0.43859	0.02134
C33	0.58412	0.41925	0.37100
C34	0.65306	0.41145	0.09539
H35	0.64576	0.45602	-0.15356
C36	0.60297	0.39160	0.43108
H37	0.55749	0.42227	0.49038
C38	0.63721	0.38821	0.29479
H39	0.68011	0.40757	-0.01292
H40	0.59181	0.37262	0.59236
N41	0.65495	0.36042	0.35729
C42	0.69281	0.35733	0.40657
H43	0.70985	0.37766	0.48296
C44	0.70774	0.32567	0.35811
C45	0.68738	0.30069	0.21933
C46	0.74400	0.31865	0.44699
N47	0.70301	0.27139	0.17108
H48	0.65911	0.30418	0.14129
C49	0.75995	0.29000	0.40638
H50	0.76205	0.33648	0.56030
C51	0.73955	0.26660	0.26745
H52	0.68767	0.74845	0.94138
C53	0.90511	0.00000	0.00000
C54	0.98105	0.00000	0.00000
H55	0.87534	0.00000	0.00000
C56	0.40520	0.50000	0.00000
C57	0.48107	0.50000	0.00000
H58	0.37546	0.50000	0.00000

Section II: Supplementary methods

1. Materials and methods

All reagents, unless otherwise noted, were purchased from commercial sources and used without further purification. n-Butanol (n-BuOH), o-dichlorobenzene (o-DCB), and acetic acid were dried through the standard procedures. Acetone, methol, tetrahydrofuran and aqueous acetic were bought from Aladdin Reagent. 1,4-dihydroxybenzidine, 1,3,6,8-Tetra(4-formylphenyl)pyrene, 2,2'-bipyridine-5,5'-dicarbaldehyde and 4,4',4'',4'''-(pyrene-1,3,6,8-tetrayl)tetraaniline was bought from Jilin Chinese Academy of Sciences-Yanshen Technology.

The Brunauer-Emmett-Teller (BET) surface areas of COFs were measured at 77 K by using a Quantachrome Automated Surface Area & Pore Size Analyzer. Pore size distributions was estimated by nonlocal density functional theory (NLDFT). The powder X-ray diffraction (PXRD) pattern was recorded on a Cu-Kα X-ray radiation source (λ=0.154056 nm) incident radiation by a Rigaku MiniFlEX 600 instrument over the range of 2θ =2.0~40.0° with a step size of 0.02° per step. The FT-IR spectra were recorded by Thermo Nicolet iS50 in the range from 400 to 4000 cm^{-1}. Solid-state ^{13}C CP/MAS NMR spectra were recorded on 400WB S2 AVANCE III (Bruker, Switzerland) plus 400 MHz spectrophotometer at 298 K. Morphological information for COFs were obtained from field-emission scanning electron microscope (SEM, FEI Nano 450), transmission electron microscopy (TEM, JEOL jem 2100f) and high-angle annular dark-field scanning transmission election microscope (HAADF-STEM, FEI Themis Z). The X-ray photoelectron spectroscopic (XPS) was obtained from an ESCALAB 250 with a monochromatic Al Kα X-ray source. Thermogravimetric analyses (TGA) was recorded on a Netzsch Model STA 449C microanalyzer heated from 25 °C to 900 °C in nitrogen atmosphere. UV-visible absorption spectra of the polymers were measured on a Shimadzu UV-2550 UV-vis spectrometer by measuring the reflectance of powders in the solid state. High-resolution valence band ultraviolet photoelectron spectra (UPS) were obtained from Thermo Fisher Scientific Escalab 250Xi. Fluorescence spectrum and fluorescence lifetime were measured using a FLS1000 Edinburgh Instruments spectrofluorimeter. The Photocatalytic hydrogen evolution measurements were carried out in a Pyrex top-irradiation reaction vessel connected to a glass closed Labsolar 6A gas circulation system (Perfect Light) and gas products were analyzed by online 8890 GC System (Agilent) referencing against standard gas with a known concentration of hydrogen. The light intensities of high precision illuminator system LX300f were tested by the PM100D optical power meter (Tech Support). The electrochemical measurements were recorded on the NOVA II electrochemical workstation with a standard three-electrode system with the photocatalyst-coated FTO as the

working electrode, Pt plate as the counter electrode and the Ag/AgCl electrode as a reference electrode.

Photocatalytic hydrogen evolution measurement

The photocatalytic hydrogen evolution measurements were carried out in a Pyrex top irradiation reaction vessel connected to a glass closed gas circulation system (Labsolar 6A, Perfect Light). For each reaction, it was conducted using 10 mg photocatalyst, 100 mL H_2O and 176 mg ascorbic acid as sacrificial agent, and the mixture was dispersed by ultrasonication for 30 minutes to obtain a uniform dispersion. After that, appropriate H_2PtCl_6 aqueous solution (0.376 g Pt L^{-1}) was added into the solution system. Then the samples were added into a quartz transparent photoreactor. The above suspension was bubbled with Argon for 30 minutes, and was kept at 20 °C using circulating water. The Xenon lamp was turned on to start the photocatalysis measurements. Besides, the reaction solution was constantly stirred to maintain the entire mixture homogeneous. Hydrogen dissolved in the reaction mixture was not measured and the pressure increase generated by the evolved hydrogen was neglected in the calculations. The hydrogen evolution rates were determined from a linear regression fit. After the photocatalysis experiment, the photocatalysts were recovered by washing with water and acetone then dried at 120 °C vacuum oven.

Long-term photocatalytic experiment

The long-term photocatalytic hydrogen evolution measurements were carried out in a same system as the photocatalytic hydrogen evolution measurements. It was conducted using 10 mg PY-DHBD-COF, 100 mL H_2O and 352 mg ascorbic acid as sacrificial agent, and the mixture was dispersed by ultrasonication for 30 minutes to obtain a uniform dispersion. After that, 3 wt% Pt (795 μL 0.376 g Pt L^{-1} H_2PtCl_6 aqueous solution) was added into the solution system. During the long-term experiment, 352 mg ascorbic acid was added for eleven times only when the amount of hydrogen no longer increased.

Apparent quantum yield (AQY) measurement

The AQY measurement was conducted in the same reaction system as other photocatalytic reactions, otherwise the xenon lamp was equiped with a band-pass filter with central wavelength of 420 nm and full-width at half-maximum (FWHM) of ~10 nm. The number of photons reaching the solution was measured using a calibrated Si photodiode. For full absorption of the incident photons, 50 mg 3% Pt-PY-DHBD-COF was used as photocatalyst in the AQY measurement. The AQY value was calculated according to the following equation:

$$\mathrm{AQY}(\%) = (2R(\mathrm{H_2}) / I) \times 100 \qquad \text{(S1)}$$

where $R(H_2)$ and I denote the evolution rate of H_2 in the initial one hour irradiation and the number of photons reaching the surface of the reaction solution per hour, repectively. The

total number of incident photons per hour were measured to be 4.3 × 10^{19} h^{-1}. The H_2 evolution rate was 3 μmol h^{-1} (this is 1.806 × 10^{18} h^{-1}).

Photoelectrochemical measurements

There are two preparations before the test: Firstly, FTO glasses were firstly cleaned by sonication in ethanol for 30 min and dried under nitrogen flow. Secondly, the working electrodes were immersed in the electrolyte for 60 s before any measurements were taken. After that, the photocurrent-time (I-t) profiles, electrochemical impedance spectra (EIS), Mott-Schottky plot were recorded on the NOVA II electrochemical workstation with a standard three-electrode system with the photocatalyst-coated FTO as the working electrode, Pt plate as the counter electrode and the Ag/AgCl electrode as a reference electrode. A 0.2 M Na_2SO_4 solution (pH = 2.5) was used as the electrolyte. A 300 W Xenon lamp with a 420 nm cut-off filter was used as the light source during the measurement. The applied potentials vs. Ag/AgCl is converted to RHE potentials using the following equation:

$$E_{\mathrm{RHE}} = E_{\mathrm{Ag/AgCl}} + 0.0591\mathrm{pH} + E^{\theta}_{\mathrm{Ag/AgCl}}\ (E^{\theta}_{\mathrm{Ag/AgCl}} = 0.199\ \mathrm{V}) \quad \text{(S2)}$$

DFT calculations

Periodic structure calculations on the COF crystal structures were carried out within the UFF[1] force field using LAMMPS code[2] considering the staking structures is too time-consuming to be calculated with DFT method. The atom type of N, O, H and C is N_R, O_R, H_ and C_R as described in the UFF force field. Steepest descent method has been used to search the lowest energy configuration.

The hole and electron distribution are calculated with equation S3-S8 using the Multiwfn code:

$$\rho^{\mathrm{hole}}(r) = \rho^{\mathrm{hole}}_{\mathrm{loc}}(r) + \rho^{\mathrm{hole}}_{\mathrm{cross}}(r) \quad \text{(S3),}$$

$$\rho^{\mathrm{hole}}_{\mathrm{loc}}(r) = \sum_{\mathrm{i}\to a}(w_i^a)^2\varphi_i\varphi_i - \sum_{\mathrm{i}\leftarrow a}(w_i'^a)^2\varphi_i\varphi_i \quad \text{(S4),}$$

$$\rho^{\mathrm{hole}}_{\mathrm{cross}}(r) = \sum_{i\to a}\sum_{\mathrm{j}\neq\mathrm{i}\to a} w_i^a w_j^a\varphi_i\varphi_j - \sum_{i\leftarrow a}\sum_{\mathrm{j}\neq\mathrm{i}\leftarrow a} w_i'^a w_j'^a\varphi_i\varphi_j \quad \text{(S5),}$$

$$\rho^{\mathrm{ele}}(r) = \rho^{\mathrm{ele}}_{\mathrm{loc}}(r) + \rho^{\mathrm{ele}}_{\mathrm{cross}}(r) \quad \text{(S6),}$$

$$\rho^{\mathrm{hole}}_{\mathrm{loc}}(r) = \sum_{\mathrm{i}\to a}(w_i^a)^2\varphi_a\varphi_a - \sum_{\mathrm{i}\leftarrow a}(w_i'^a)^2\varphi_a\varphi_a \quad \text{(S7),}$$

$$\rho^{\mathrm{hole}}_{\mathrm{cross}}(r) = \sum_{i\to a}\sum_{\mathrm{i}\to b\neq a} w_i^a w_i^b\varphi_a\varphi_a - \sum_{i\leftarrow a}\sum_{\mathrm{i}\leftarrow b\neq a} w_i'^a w_i'^b\varphi_a\varphi_a \quad \text{(S8),}$$

where ρ^{hole}(r) and ρ^{ele}(r) stand for the density distribution of hole and electron, respectively, r is the distance, φ is the orbital wave function, φ_i or φ_j is the occupied orbital, and φ_a or φ_b is the unoccupied orbital, and w and w' correspond to configuration coefficient of excitation and

de-excitation, respectively. Therefore, i→a represents excitation configuration, i←a represents de excitation configuration. Hole distribution and electron distribution are divided into local term and cross term. The local term is generally dominant, reflecting the contribution of the configuration function itself, and the cross term can not be ignored, otherwise the quantification is inaccurate, which reflects the influence of the coupling between the configuration functions on the hole and electron distribution.

S and D indexes stand for the overlap integral of hole-electron distribution and the distance between centroid of hole and electron, respectively. The S and D are calculated with equations S9-S10 using Multiwfn code:

$$S = \int \min\left[\rho^{\text{hole}}(r), \rho^{\text{ele}}(r)\right] dr \quad \text{(S9)},$$

$$D = \sqrt{\left|X_{ele} - X_{hole}\right|^2 + \left|Y_{ele} - Y_{hole}\right|^2 + \left|Z_{ele} - Z_{hole}\right|^2} \quad \text{(S10)},$$

where $\rho^{hole}(r)$ and $\rho^{ele}(r)$ stand for the density distribution of hole and elect
ron, respectively, X_{hole} refers to the X coordinate of centroid of hole, which can be obtained through multiplying the p^{hole} function by the x coordinate variable and integration in the whole space. The detailed settings and process can be found in part 4.18 "Electron excitation analysis" in the manual of Multiwfn code (Tian Lu, Multiwfn Manual, version 3.8, Section 4.18, available at http://sobereva.com/multiwfn/misc/Multiwfn_3.8.pdf).

2. Synthetic procedures

PY-DHBD-COF. A n-butylalcohol (n-BuOH)/o-dichlorobenzene (o-DCB)/6 M AcOH (5/5/1 by vol.; 1.1 mL) mixture of 1,4-dihydroxybenzidine (0.04 mmol, 8.6 mg) and 1,3,6,8-tetraphenylpyrene (0.02 mmol, 12.3 mg) in a Pyrex tube (10 mL) was degassed by three freeze-pump-thaw cycles. The tube was sealed off and heated at 120 °C for 3 days. The precipitate was collected by centrifugation, and washed with anhydrous THF for 5 times and acetone twice. The powder was dried at 120 °C under vacuum overnight to give the PY-DHBD-COF in an isolated yield of 85%.

PY-BPY-COF. A n-butylalcohol (n-BuOH)/o-dichlorobenzene (o-DCB)/6 M AcOH (5/5/1 by vol.; 1.1 mL) mixture of 2,2'-bipyridine-5,5'-dicarbaldehyde (2,2'-BPY) (0.04 mmol, 8.5 mg) and 4,4',4'',4'''-(pyrene-1,3,6,8-tetrayl) tetraaniline (PY-NH_2) (0.02 mmol, 11.3 mg) in a Pyrex tube (10 mL) was degassed by three freeze-pump-thaw cycles. The tube was sealed off and heated at 120 °C for 3 days. The precipitate was collected by centrifugation, and washed with anhydrous THF for 5 times and acetone twice. The powder was dried at 120 °C under vacuum overnight to give the PY-BPY-COF in an isolated yield of 82%.

Supplementary References

1. Rappe, A. K., Casewit, C. J., Colwell, K. S., Goddard, W. A. & Skiff, W. M. UFF, a full periodic-table force-field for molecular mechanics and molecular-dynamics simulations. *J. Am. Chem. Soc.* **114**, 10024-10035 (1992).
2. "LAMMPS Molecular Dynamics Simulator". Sandia National Laboratories. Retrieved 2010-10-03.
3. Mi, Z. *et al.* Covalent organic frameworks enabling site isolation of viologen-derived electron-transfer mediators for stable photocatalytic hydrogen evolution. *Angew. Chem. Int. Ed.* **60**, 9642-9649 (2021).
4. Chen, W. *et al.* Modulating benzothiadiazole-based covalent organic frameworks via halogenation for enhanced photocatalytic water splitting. *Angew. Chem. Int. Ed.* **59**, 16902-16909 (2020).
5. Wang, X. *et al.* Sulfone-containing covalent organic frameworks for photocatalytic hydrogen evolution from water. *Nat. Chem.* **10**, 1180-1189 (2018).
6. Bi, S. *et al.* Two-dimensional semiconducting covalent organic frameworks via condensation at arylmethyl carbon atoms. *Nat. Commun.* **10**, 2467 (2019).
7. Xiao, J. D., Han, L., Luo, J., Yu, S. H. & Jiang, H. L. Integration of plasmonic effects and schottky junctions into metal-organic framework composites: steering charge flow for enhanced visible-light photocatalysis. *Angew. Chem. Int. Ed.* **57**, 1103-1107 (2018).
8. Chen, Y.-F. *et al.* Calix[4]arene based dye-sensitized Pt@UiO-66-NH_2 metal-organic framework for efficient visible-light photocatalytic hydrogen production. *Appl. Catal. B* **206**, 426-433 (2017).
9. Mohammadnezhad, F. *et al.* Tuning the optoelectronic properties of hybrid functionalized MIL-125-NH_2 for photocatalytic hydrogen evolution. *ACS Appl. Mater. Interfaces* **13**, 5044-5051 (2021).
10. Li, X. *et al.* BiOI particles confined into metal-organic framework NU-1000 for valid photocatalytic hydrogen evolution under visible-light irradiation. *Inorg. Chem.* **60**, 1352-1358 (2021).
