# Supplementary material for: In situ photodeposition of platinum clusters on a covalent organic framework for photocatalytic hydrogen production
Source: Nat Commun. 2022 Mar 15;13:1355. doi: 10.1038/s41467-022-29076-z (PMC8924255; doi:10.1038/s41467-022-29076-z)
Supplement: Supplementary file 3 — Description of Additional Supplementary Files [file 41467_2022_29076_MOESM3_ESM.pdf]

Description of Additional Supplementary Files

File name: Supplementary Data 1

Description: The relevant DFT optimized structures
